# Adaptation of a Problem-solving Program (Friendship Bench) to Treat Common Mental Disorders Among People Living With HIV and AIDS and on Methadone Maintenance Treatment in Vietnam: Formative Study

**DOI:** 10.2196/37211

**Published:** 2022-07-08

**Authors:** Ha V Tran, Ha T T Nong, Thuy T T Tran, Teresa R Filipowicz, Kelsey R Landrum, Brian W Pence, Giang M Le, Minh X Nguyen, Dixon Chibanda, Ruth Verhey, Vivian F Go, Hien T Ho, Bradley N Gaynes

**Affiliations:** 1 The University of North Carolina Vietnam Office Hanoi Vietnam; 2 Faculty of Environmental and Occupational Health Hanoi University of Public Health Hanoi Vietnam; 3 Department of Epidemiology University of North Carolina Chapel Hill, NC United States; 4 Center for Research and Training in HIV/AIDS Hanoi Medical University Hanoi Vietnam; 5 Faculty of Epidemiology Department Hanoi Medical University Hanoi Vietnam; 6 Department of Community Medicine & Research Support Centre University of Zimbabwe Harare Zimbabwe; 7 London School of Hygiene & Tropical Medicine London United Kingdom; 8 Department of Health Behavior, Gillings School of Global Public Health University of North Carolina Chapel Hill, NC United States; 9 Faculty of Clinical Medicine Hanoi University of Public Health Hanoi Vietnam; 10 Department of Psychiatry University of North Carolina Chapel Hill, NC United States

**Keywords:** Friendship Bench, Vietnam, Assessment-Decision-Adaptation-Production-Topical Experts-Integration-Training-Testing, ADAPT-ITT, common mental disorders, people living with HIV, PWH, people who inject drugs, PWID, methadone maintenance treatment, MMT, depression, anxiety, stress disorder

## Abstract

**Background:**

The prevalence of common mental disorders (CMDs) among people living with HIV and people who inject drugs is high worldwide and in Vietnam. However, few evidence-informed CMD programs for people living with HIV who inject drugs have been adapted for use in Vietnam. We adapted the *Friendship Bench* (FB), a problem-solving therapy (PST)–based program that was successfully implemented among patients with CMDs in primary health settings in Zimbabwe and Malawi for use among people living with HIV on methadone maintenance treatment (MMT) with CMDs in Hanoi, Vietnam.

**Objective:**

This study aimed to describe the adaptation process with a detailed presentation of 4 phases from the third (adaptation) to the sixth (integration) of the Assessment-Decision-Adaptation-Production-Topical Experts-Integration-Training-Testing (ADAPT-ITT) framework.

**Methods:**

The adaptation phase followed a qualitative study design to explore symptoms of CMDs, facilitators, and barriers to conducting FB for people living with HIV on MMT in Vietnam, and patient, provider, and caretaker concerns about FB. In the production phase, we revised the original program manual and developed illustrated PST cases. In the topical expert and integration phases, 2 investigators (BNG and BWP) and 3 subject matter experts (RV, DC, and GML) reviewed the manual, with reviewer comments incorporated in the final, revised manual to be used in the training. The draft program will be used in the training and testing phases.

**Results:**

The study was methodologically aligned with the ADAPT-ITT goals as we chose a proven, effective program for adaptation. Insights from the adaptation phase addressed the who, where, when, and how of FB program implementation in the MMT clinics. The ADAPT-ITT framework guided the appropriate adaptation of the program manual while maintaining the core components of the PST of the original program throughout counseling techniques in all program sessions. The deliverable of this study was an adapted FB manual to be used for training and piloting to make a final program manual.

**Conclusions:**

This study successfully illustrated the process of operationalizing the ADAPT-ITT framework to adapt a mental health program in Vietnam. This study selected and culturally adapted an evidence-informed PST program to improve CMDs among people living with HIV on MMT in Vietnam. This adapted program has the potential to effectively address CMDs among people living with HIV on MMT in Vietnam.

**Trial Registration:**

ClinicalTrials.gov NCT04790201; https://clinicaltrials.gov/ct2/show/NCT04790201

## Introduction

Injection drug use is the main cause of the HIV epidemic in Vietnam, resulting in a high HIV prevalence among people who inject drugs, 15% in 2017 [1] and 12.7% in 2020 [2]. Antiretroviral therapy (ART) and methadone maintenance treatment (MMT) have been implemented in Vietnam since 2004 and 2008, respectively, and people living with HIV who inject drugs are commonly treated with simultaneous ART and MMT [3,4]. HIV and drug addiction are chronic stressors on mental health, and when combined with many other barriers related to HIV and drug addiction such as stigma and discrimination, the prevalence of common mental disorders (CMDs) among people living with HIV who inject drugs is higher than in the general population [5,6]. CMD is a collective term that refers to a range of depressive, anxiety, and stress-related disorders [7]. Depression is characterized by sadness, low self-esteem, tiredness, lack of concentration, lack of interest, and sleeping difficulties. Anxiety disorders refer to the feeling of anxiety, fear, panic including posttraumatic disorder, and social anxiety [7]. Stress is a state of physical and emotional tension caused by reactions to stressful stimuli from life and the environment [8]. The prevalence of depression and anxiety disorder among MMT clients in China in 2017 was 12.8% and 19.5% [9], compared with only 4.4% and 3.6% in the general population in 2015, respectively [7]. In a study by Levintow et al [10], people living with HIV who inject drugs in North Vietnam had a mild depression rate of 25% and moderate depression rate of 44%. Mughal et al [11] reported that people living with HIV on MMT in Hanoi had CMDs of depression and anxiety and posttrauma stress rate of 17% [11]. Prior research has demonstrated the effectiveness of behavioral and mental health programs among people living with HIV [12], including an evidence-informed program (EIP) to decrease levels of distress among people living with HIV in Thai Lan [13], an adapted life step intervention to effectively improve depression among people living with HIV in Zimbabwe [14], and an adapted CMD program for people living with HIV proved effective in 2020 in Malawi [15]. In Vietnam, we found evidence that family group counseling improved depression symptoms among people who inject drugs in 2017 [16]. Other studies have also recommended the need for CMD programs for people who inject drugs in Vietnam [17]. Problematically, there have been no mental health programs for this population and setting.

*Friendship Bench* (FB), developed by Chibanda Dixon in 2006 [18], is a brief evidence-informed psychological program using problem-solving therapy (PST) and implemented by lay counselors. More than 50,000 people have received counseling in the FB program, making it the largest mental health program integrated into primary health care in Africa, supported by the government and the Ministry of Health of Zimbabwe [18]. FB has been validated through a clinical trial to reduce CMDs among community members in Zimbabwe. In this study, people receiving FB sessions had lower depression and anxiety scores on the Shona Symptoms Questionnaire (SSQ-14) than those who did not receive FB sessions (3.81 and 8.90, respectively). Moreover, the percentage of people in the intervention arm who had depression symptoms on the Patient Health Questionnaire-9 was lower than that in the control arm (13.7% in the intervention arm compared with 49.9% in the control arm) [19]. FB was used in the provision of ART adherence and depression counseling sessions to people living with HIV in Malawi. A total of 501 people living with HIV who had just started ART and had mild depression (Patient Health Questionnaire-9 scores from 5 to 9) were randomized into 2 groups: the intervention group and the control group. The study indicated that people living with HIV in the intervention group had a higher rate of adherence to ART than those in the control group [15]. Recently, FB had been adapted to reduce depression and anxiety among people living with HIV adolescents in a clinical trial in Zimbabwe. The study of this adaptation is ongoing [20]. FB is also currently being adapted for use among people living with HIV who are pregnant in Malawi [21].

FB consists of up to 6 structured 45-minute sessions, and it has been effective in treating CMDs among community members in Zimbabwe [19,22]. The sessions were conducted outside of the primary clinics. Each session followed a PST approach and included steps to determine a manageable problem, choose a problem to solve, make a plan to solve the problem, create a follow-up of plan implementation, and encourage work on new problems. After completing an individual session, participants are invited to take part in group activities, where people facing similar life challenges share their stories, spend time together, and work on income-generating activities [22]. The original FB manual has 23 chapters, covering the topics of counseling techniques and session structure, core competencies of counselors, mental illness, HIV and mental health, substance use disorders (SUD), and PST. However, to implement this program for a new population, we first simplified and restructured the program to meet local norms [23], the new target population, and the new study settings [24] via the Assessment-Decision-Adaptation-Production-Topical Experts-Integration-Training-Testing (ADAPT-ITT) model [25].

The adaptation method ADAPT-ITT of the Centers for Disease Control and Prevention (CDC) was developed by Wingood and DiClemente in 2008 as a framework to guide the adaptation of a proven EIP to the specific goals and study participants of HIV prevention [25]. The ADAPT-ITT model has been used to adapt HIV prevention EIP for HIV key populations such as African American couples in the United States [26], people living with HIV in Zimbabwe [27], African American women in the United States [28], and men who have sex with men in Thailand [29] and to address mental and sexual health for young people living with HIV in sub-Saharan Africa [30].

This study is nested within the parent study “Adaptation of the Friendship Bench counseling programme to improve mental health and HIV care engagement outcomes among people living with HIV who inject drugs in Vietnam,” which aimed to test the feasibility, acceptability, and fidelity of the adapted FB in Vietnam [31]. This paper describes the process of FB adaptation to address CMDs among people living with HIV on MMT in some MMT clinics in Hanoi, Vietnam, in 2021, in preparation for a randomized controlled trial (RCT) following the ADAPT-ITT framework and specifically focuses on ADAPT-ITT phases 3 to 6.

## Methods

### Overview

The ADAPT-ITT model [25] comprises 8 sequential phases. Phase 1, the assessment, was conducted in a previous study [17]. Phase 2, the decision, was conducted by the parent study [31]. This paper describes in detail from the third to the sixth phases of the model: phase 3 (adaptation), phase 4 (production), phase 5 (topical experts), and phase 6 (integration). Phase 7 (training) and phase 8 (testing) will be presented in future papers.

### Phase 1: Assessment

This phase involved needs assessment of people living with HIV on MMT for reduction in CMDs. The study conducted 28 in-depth interviews (IDIs) with people living with HIV who inject drugs (n=16, 57%), HIV and MMT providers (n=8, 29%), and health care providers (n=4, 14%) in private clinic rooms in Hanoi, Vietnam in May 2018.

Results of the study showed that both health care providers and people living with HIV who inject drugs believed that people living with HIV who inject drugs were particularly susceptible to CMDs, especially depression, and had a high need for mental health treatment. The study also recommended integrating mental health care into MMT clinics to increase mental health access to people living with HIV on MMT [17].

### Phase 2: Decision

This phase involved reviewing evidence-informed CMD programs and deciding which EIP to select for the new target population. Few programs addressing CMDs for people living with HIV exist in low- and middle-income countries, which have limited mental health resources. One of the few evidence-informed CMD counseling programs is the “FB” model, which has extensive validation in low- and middle-income countries [19]. FB helped to reduce CMDs, which was the aim of this study. In addition, FB has also been adapted for people living with HIV in Zimbabwe and Malawi [15,20,21] and may be suitable for people living with HIV on MMT in Vietnam.

### Phase 3: Adaptation

In phase 3, stakeholders including people living with HIV on MMT, their family members, and health care providers reviewed the original FB content and implementation methods to answer questions related to who, when, where, and how to deliver FB in Vietnam for appropriate implementation in MMT clinics. The study conducted IDIs with 12 people living with HIV on MMT, 5 family members and caretakers of the patients, and 4 clinic directors. In all, 2 focus group discussions (FGDs) were conducted with MMT health care providers (each group had 5 people).

Inclusion criteria for patients were being aged ≥18 years, willing to participate in the study, and having at least one of the symptoms: depression, anxiety, stress disorders as indicated by the Depression, Anxiety, and Stress Scale-21 items (DASS-21), consisting of 21 questions and 3 items measuring levels of the emotional states of depression, anxiety, and stress. The DASS-21 is based on a dimensional conception of depression, anxiety, and stress as part of the full human experience rather than the categorical approach of either having a disorder or not (as used in the Diagnostic and Statistical Manual of Mental Disorders or International Classification of Diseases) [32]. Although FB was initially tested using the SSQ-14 to screen for CMDs, this tool has not been validated in Vietnam. The DASS-21, however, has been validated in this setting. Originally developed by Lovibond [32] in Australia in 1983 and tested to screen for CMDs in the community, the DASS-21 has been translated and successfully validated for a variety of study participants in Vietnam, including adolescents [33], rural women [34], health care workers [35], and people living with HIV on MMT [17]. The DASS-21 is aligned with the SSQ-14 in terms of screening CMDs, including depression and anxiety disorders in the general population, but is distinguished by having a stress disorder scale. Thus, the study team chose the DASS-21 as a research tool, as it has the advantages of validity in this population, brevity, ease of implementation, and measurement of depression, anxiety disorder, and stress disorder relevant to the purpose of the study. The study will also use suicide risk assessment that was effectively used in the HPTN074 study for people living with HIV who inject drugs in Vietnam [36].

Inclusion CMDs scores were at least a moderate level in which depression subscale score was ≥14, anxiety subscale score was ≥10, and stress subscale score was ≥19. People who were aged <18 years, did not meet the DASS-21 moderate score, had cognitive impairments, or had suicidal thoughts (exclusion, therefore, included referral for standard clinical follow-up care) were excluded from the study. Patients who met the inclusion criteria were invited to participate in an IDI. Family members and caretakers were the patients’ parents, spouses, or main caretakers who already knew the patients’ HIV and MMT status and who were introduced by the patients. Only family members and caretakers who had patients’ consent to approach and who already knew the patients’ HIV and MMT status were invited for IDIs. The inclusion criteria for health care providers were having at least two years of counseling and clinical experience with people living with HIV and people who inject drugs.

IDI and FGD guides were developed in English in accordance with previous FB interviews and the objectives of this study. The interview guides were reviewed for linguistic and cultural appropriateness. The guides were translated into Vietnamese, discussed, and revised by the research team, who had knowledge and experience on the study topic and participants. The guides were piloted among 5 patients who met the study inclusion criteria but received treatment from another MMT clinic and did not participate in the study. The IDIs and FGDs were facilitated in Vietnamese by 2 interviewers who were trained in qualitative research methods and had extensive experience working with people living with HIV who inject drugs. Each IDI had an average duration of 50 (SD 18) minutes. The average FGD duration was 1.5 (SD 25) hours. All IDIs and FGDs were conducted in Vietnamese, audio recorded, and transcribed verbatim. The interview and FGD transcriptions were deidentified and translated into English. The analysis was conducted according to the applied thematic analysis approach [37]. Thematic analysis was performed using NVivo (version 12.0; QSR International). All qualitative research was guided by the consolidated criteria for reporting qualitative studies), a 32-item checklist [38]. In all, 4 study members with experience in qualitative data analysis were involved in the data analysis process. The list of parent and child codes was developed and agreed upon by the coders. Parent and child codes and definitions were created a priori and were derived from the interview guide and literature review. Furthermore, 20% of the transcripts were double coded. Intercoder reliability was assessed after all coders coded the first 2 transcripts. A total of 2 meetings were held among the coders to discuss the coding results item by item. The official coding process began after all 4 coders agreed on the coding method and procedure. The codebook was refined throughout the analysis process and the findings were presented, discussed, and agreed upon by the study team.

### Phase 4: Production

In all, 3 external content experts reviewed the phase 3 results. The original FB manual study procedures and content were modified according to expert comments. On the basis of the content experts’ review, the manual was tailored for use by both health care providers and lay counselors. In addition, the experts’ feedback informed the study team to include relevant matters regarding injecting drugs and HIV transmission in Vietnam. Finally, all the case studies were revised to make them suitable for participants in Vietnam.

### Phases 5 and 6: Topical Experts and Integration

We solicited and integrated input and feedback on the first draft of the adapted FB program from 2 investigators, 2 creators of FB, and 1 topical expert on behavioral programs for people living with HIV and SUD from Hanoi Medical University (HMU). This feedback informed the development of the next draft of the FB manual. Culturally, Vietnamese illustrations were designed to replace Zimbabwean illustrations and were added to the second manual draft in English. A total of 2 translators of the study team had experience with people living with HIV who inject drugs and mental health and were responsible for translating the second draft. The Vietnamese topical expert revised the translated draft to ensure conceptual equivalency. The team then revised the translation again and agreed on the second FB manual version.

### Phases 7 and 8: Training and Testing

Using the second adapted version, the creators of FB led a Training of Trainers session for 6 study team members in Vietnam and 3 people from HMU via a 5-day virtual training course. Trainers in Vietnam then provided in-person 5-day training for lay counselors and health care counselors in MMT clinics. The second draft was then piloted with 5 eligible patients to further refine the session content. The process culminated in a final version of the adapted FB manual that would be ready for formal evaluation in an RCT study (Table 1).

**Table 1:** The Assessment-Decision-Adaptation-Production-Topical Experts-Integration-Training-Testing (ADAPT-ITT) model [25] applied in the study.

Phase	Method	Version
1: Assessment of new study population	Assessed needs of people living with HIV on MMT^a^ for CMDs^b^	Completed in 2019
2: Decision on choosing EIP^c^ for adaptation	Reviewed EIPs for CMDs then decided to choose FB^d^ counseling CMDs for community people of Zimbabwe to adapt for counseling CMDs for people living with HIV on MMT	Completed in 2019
3: Adaptation	Conducted IDI^e^ with 12 people living with HIV on MMT, 5 family members and caretakers, 4 clinic directors, and 2 FGDs^f^ with MMT health care providers (each group had 5 people); analyzed results by themes and reported results	Original manual
4: Production	Presented results of phase 3 to 2 creators of FB and 2 investigators and 1 expert in HMU^g^; recorded and reported comments of 2 investigators and topical experts for production; reviewed original FB manual to modify per results above and received investigators’ agreement on adaptation into the first draft manual	First draft
5: Topical experts	Sent the first draft manual to 3 topical experts and 2 investigators for comments and revision	First draft
6: Integration	Integrated experts’ and investigators’ comments to make the second draft, added Vietnamese illustrations to make the third draft in English, and sent the third draft to experts and investigators again for comments; translated the third draft to Vietnamese; sent the Vietnamese version of the third draft to a Vietnamese topical expert for revision and to ensure conceptual equivalency	Second draft
7: Training	Creators of FB trained for trainers in Vietnam; trainers trained for counselors	Third draft
8: Testing	Piloted the third draft for 5 people living with HIVs on MMT and having CMDs; revised third draft to make final manual	Final

^a^MMT: methadone maintenance treatment.

^b^CMD: common mental disorder.

^c^EIP: evidence-informed program.

^d^FB: Friendship Bench.

^e^IDI: in-depth interview.

^f^FGD: focus group discussion.

^g^HMU: Hanoi Medical University.

### Ethics Approval

The study protocol is available at ClinicalTrials.gov (NCT04790201). The study protocol, interview guides, and informed consent forms were approved by the institutional review boards at University of North Carolina at Chapel Hill on August 24, 2020 (study 20-1689), and HMU on June 19, 2020 (decision 119 ĐHYHN). All the study participants provided written informed consent in Vietnamese.

## Results

### Phase 3: Adaptation

#### Overview of the Study Participants

We approached 67 patients using purposive sampling representing people living with HIV on MMT in 4 MMT clinics in Hanoi to answer the DASS-21 questions. Less than one-third of the patients approached (19/67, 28%) met the DASS-21 threshold CMD scores. Of 19 eligible patients, 12 (63%) agreed to be interviewed. A patient was female. The average age of the patients was 44 years. In all, 83% (10/12) of the patients had symptoms of depression, 83% (10/12) had symptoms of anxiety, and 33% (4/12) had symptoms of stress at screening (Table 2).

The age of family members and caretakers ranged from 60 to 73 years. All participants were either retired or unemployed. Health care providers included 2 MMT clinic directors, 1 associate director of CDC Hanoi, and 1 deputy director of the Hanoi Department of Health. A total of 10 health care workers, including MMT physicians and nurses in MMT clinics aged 35 to 66 years, participated in 2 FGDs.

The adaptation phase in our study aimed to explore the most culturally appropriate way to adapt FB in Vietnam. Using IDIs with patients, their family members or caretakers, clinic directors, and FGDs with health care providers, we addressed the who, where, when, and how regarding the administration of the FB program in the MMT clinics.

**Table 2:** Demographic characteristics of participants (N=12).

Demographic characteristics			Value
**Gender, n (%)**			
	Male	11 (92)	
	Female	1 (8)	
Age (years), mean (SD; range)			44 (6; 35-56)
**Employment, n (%)**			
	Unemployed	6 (50)	
	Working full-time or part-time	6 (50)	
**Marital status, n (%)**			
	Single	5 (42)	
	Married	3 (25)	
	Divorced or separated	4 (33)	
Years on ART^a^, mean (SD)			8 (6)
Years on MMT^b^, mean (SD)			5 (2)
**DASS-21^c^, n (%)**			
	Symptom of depression	10 (83)	
	Symptom of anxiety	10 (83)	
	Symptom of stress	4 (33)	
**Number of types of different CMD^d^ symptoms, n (%)**			
	One symptom	3 (25)	
	Two symptoms	6 (50)^e^	
	Three symptoms	3 (25)	

^a^ART: antiretroviral therapy.

^b^MMT: methadone maintenance treatment.

^c^DASS-21: Depression, Anxiety, and Stress Scale-21 items.

^d^CMD: common medical disorder.

^e^Overall, 5 had depression and anxiety 1 had anxiety and stress.

#### Who Should Deliver the Program

When being asked who (health staff person or peer) should provide the FB program for people living with HIV on MMT, the study respondents gave mixed reviews of who would be best for the FB program. Broadly, it appeared that directors and focus group respondents saw more advantages with a staff member as the program provider. Among family members, caretakers, and patient respondents, there was no distinct, overarching opinion on a preferred program provider. A total of 2 directors and 2 patient respondents shared that the professionalism of health care providers would be beneficial. A family member stressed that staff members should be purposively selected and need certain personality traits to be as effective as lay counselors, including honesty and willingness to share their experiences with patients in FB. Clinic providers noted that clinical experience with ART and MMT management are also helpful skills for a potential program provider. They shared that their mental health counseling experience may not be at the level needed for this type of program, as exemplified in focus group FGD_01:

It’s certainly good. We haven’t been trained in depression. We haven’t had a chance to attend any depression courses. That’s why we can’t help our patients. We just told the patient’s family to take the patient to the psychiatric clinic. Without experience, we can’t support more than that. I have worked here for many years, but there has been no training course for depression.

A director emphasized key aspects to improve and that staff members as program providers may need further training and understanding, enthusiasm, and gentleness:

Knowledge is always along with skills. They must be trained in skills and knowledge, when they understand, they can do it. Their attitude and their speech towards the patients must be from their hearts. If it’s a real feeling, the patients will believe in the health workers. Patients are sensitive, sometimes only a glance could affect them.

Director_IDI_D03

For the program led by peers, participants from different groups (1 director, 4 FGD participants, 2 family members, and 7 patients) agreed that peers could understand patients very well. Peers and patients had many similarities in terms of life, health, and living conditions. Peers understood the patient’s language and experienced similar things, both good and bad. In addition, formality was unnecessary between them, making it easier for patients to talk and share their opinions with peers, which could not be achieved with staff members:

I think it’s feasible because they are the persons same as us, so they can understand the mindset of sick people like me, you are not sick so you cannot understand our thoughts.

Patient_IDI_102

Moreover, peers had a better understanding of patients’ health conditions from their perspectives and experiences. All of these built more trust between peers and patients, facilitating the counseling sessions. However, according to clinic providers and directors, to successfully implement the FB program, peers had to have a serious attitude and commitment to the task. In doing so, they need training on how to provide counseling and care for patients to fulfill the tasks of a counselor. In addition to their practical experience, peers should update their knowledge about the topic and improve communication skills to effectively transfer what they have learned to the patients. Only 1 clinic health care provider and 1 patient were concerned that peers might not be able to fully understand their patients’ conditions. Because of their addiction and medication and their mental state, it might be difficult to find enthusiastic and suitable peers to lead the program:

Because a peer can change differently day by day, it’s difficult to find an appropriate methadone peer, but it is easier to find an ART peer. In my clinic, there is a peer who also counsels other patients...But we should think carefully about selecting methadone peers, we should consider medical staff doing the programme because we don’t know if they [peers] are stable or not. We may have to change to others if we choose them.

FGD_01

In addition, although peers might receive training to do the job, they lack practical experience in caring for patients with different types of mental problems. A dominant theme among the patient respondents was the importance of the personality traits and counseling approach of the program provider. Patient respondents stressed the need for counselors who are gentle, empathetic, and understanding:

Someone like me needs affection, gentle speech, even a slightly unappropriated attitude will lead to my rejection. I need them to be considerate and gentle.

Patient_IDI_303

Considering the insights from interviews and FGDs in the adaptation phase, the study selected health care providers and people living with HIV or community members who were trusted by the study participants to work as study counselors in the RCT.

#### When to Deliver the Program

All clinic directors, health care providers, family members, and 9 participants (14 references from clinic directors, 15 references from FGDs, 10 references from family members, and 46 references from patients) reported their concerns about what time to implement the FB program. They all shared that as many patients work, it might be challenging to find a suitable time for them to attend the FB program. Most of the patients suggested that the counseling sessions should be in the morning when they come to take methadone:

Because we usually take the medicine in the morning and after that, we can do activities. In the afternoon, we can’t wait, at that time we have to go home, so the most convenient time is the morning.

Patient_IDI_301

A patient shared that many patients could not sit for a long time, which would affect their participation in the counseling sessions. The patient believed that the counseling time should be arranged to suit the patient’s employment and examination time, such as on weekends. For clinic directors and health care providers, apart from patients’ employment, the timetable for clinic providers is very strictly scheduled; thus, time arrangement for the clinic providers to join the FB program is problematic. Providers thought that it is best to conduct FB during office hours to ensure convenience and safety for counselors and patients:

If we do it outside of office hours, we should consider the safety of people here. If something happens, we are also affected. So we should do counseling in the daytime, not nighttime because we can’t control if they do something bad or not.

FGD_01

However, it is necessary to arrange counseling time in advance so that clinic staff do not have many overlapping tasks and have enough time for counseling patients:

So if we have such a programme then we have to have a better management of our time so that we can do it.

FGD_01

#### Where to Deliver the Program

All directors and most health care providers said that the place of program should be at the MMT clinic, as this is the place MMT patients visit every day. At MMT clinics, there were examination rooms; therefore, confidentiality and privacy would be ensured during the counseling sessions. Counseling outside the MMT clinic might be noticed by community members, which patients feared would cause them to be disliked. In addition, it is illegal for drug addicts to congregate in public spaces:

It’s not possible to gather in the community. It is against the law, they are already drug addicts, if they gather that is against the law, and people in the community don’t like that, so the methadone treatment facilities are the most suitable places. In the community, there are also a few places like that, but I only see their family come, I never see them [patients/people on treatment]. So, the medical facilities are still the best place.

Director_IDI_D03

Half of the patients (6/12, 50%) and almost all family members (4/5, 80%) preferred to receive counseling in MMT clinics for convenience and safety for both counselors and patients, ensuring privacy for patients and having timely and relevant health services if needed. The other respondents expressed that they needed to have a private and confidential place for counseling. They also suggested that the program only needs to designate a random room in the clinic as the counseling room and that it would be great if the room could have materials or visual aides to refer to or use.

#### What and How to Deliver the Program

All 4 directors mentioned that the program should be facilitated by the medical leadership. According to the directors and health care providers, to have the FB program implemented, it is crucial that the program follows all the administrative procedures of the MMT clinics before starting it. It should have a clear and detailed work plan, in which who, how, where, and when to conduct each activity are specified. Importantly, it is necessary to know about the people living with HIV on MMT and the severity of their CMD symptoms to have appropriate approaches and program sessions for them:

If the programme is implemented in the clinic, in general, in terms of administrative procedures, I also said earlier that there must be a direction. As for the implementation, first, you must have purposes and goals, then it is necessary to train staff, then find the target group we need to consult, and then plan a schedule for specific activities, jobs.

Director_IDI_01

From the perspective of patients and their family members, though they believed that patients would benefit from a program such as FB, they thought the patients would participate in the program if they received compensation for their time and effort in participating in FB sessions (3/31, 10%) and did not have to pay or have any additional restrictions for program participation (2/31, 6%):

If only he participates without any condition from the programme, that’s very good.

Family_IDI_1011

Participants thought that not having any additional challenges related to employment (10/31, 32%), financial issues (12/31, 39%), and transportation (2/31, 6%) would facilitate participation. Patients expected the program to be new and be organized interestingly and attractively:

I can’t imagine it yet, but I want new and interesting methods. We will love it and if the programme have people like you, we will join.

Patient_IDI_101

Family members and patients discussed the following recommendations: the program materials should be easy to understand; fruits, cake, and drinks should be provided; patients who actively participated in the program activities and adhered to treatment should be rewarded and praised; and if possible, the program could assist patients with job opportunities or friend-making.

Both clinic providers and patients agreed that they preferred to delicately, rather than directly, discuss mental health to help patients feel comfortable. A health care provider suggested the use of words and language that is familiar to patients to help them feel more comfortable joining the program:

I think their language when talking together is very important, they want to release and use their language to feel comfortable but they can’t do that when talking to medical staff.

FGD_01

A patient even suggested excluding the name of the program if possible. None of the suggestions for the language surrounding FB included “mental illness” in the program name.

From the perspective of family members, words in Vietnamese that are simple and easy to understand with positive meaning were preferable, and sensitive words that might worsen patients’ mental state should be avoided. A health care provider and a patient thought that the name “Friendship Bench” was beautiful and acceptable, as it did not mention the problem and had a pleasant feeling:

The Friendship bench name is also very beautiful and good.

FGD_F01

### Phase 4: Production

The results of the qualitative research in phase 3 were presented in a web-based meeting with 2 investigators, 2 creators of FB, and 1 expert from HMU to reach a consensus on the necessary adaptation of the FB manual. The original FB was adapted for use by both health care providers and lay counselors in Vietnam. Table 3 describes the details of FB adaptation. Overall, the manual illustrations of counselors were changed from Zimbabwean to Vietnamese and included both sexes. The illustrations of case studies have also been redrawn. The title “Lay Health Workers” was changed to “Counselors” to suit both groups of health care counselors and lay counselors for the study in Vietnam. The training curriculum was shortened from 8 to 5 days, and the new training program was written in a web-based format. The preface cited additional information about HIV infection and substance use patterns in Vietnam and provided a brief introduction to the adaptation of the FB manual to the study in Vietnam.

We retained the content of 8 out of the 23 chapters in the original manual. The 8 chapters included PST, counseling skills, FB cards, emotions, stabilization, strong emotional reactions, men’s health-seeking behaviors, and self-care. We completely removed 6 of the 23 chapters, namely epilepsy, belief in supernatural powers, psychosis, home visits, group circle, and proverbs. We edited the contents in 8 of the 23 chapters. Key edits included using the words *Common Mental Disorders* to refer to a collective term of depression, anxiety, and stress-related disorders instead of “kufungisisa” (Zimbabwean for depression and anxiety), replacement of SSQ-14 to DASS-21, and suicide risk assessment and management procedures, which were used in the study in Vietnam. In addition, we excluded sessions on abuse of bronchodilators, alcohol and pregnancy, and information about antipsychotics, as they were not related to the study population. Information about the use of FB on tablets was removed, as the Vietnam FB program used paper forms to record counseling session information. Information on HIV transmission through injection, substance abuse disorders caused by injecting drugs, and amphetamine-type stimulants, which were common issues of people who inject drugs—the study population in the RCT—was added.

**Table 3:** Summary of changes in Friendship Bench (FB) manual.

Chapter	Name of chapter	Revision
N/A^a^	Cover page	Changed the illustration from Zimbabwean to Vietnamese Changed the word “Lay health workers” to “Counselors” to suit both groups of counselors of the study in Vietnam
N/A	Introduction	Rewrote the preface to fit the purpose of the program in Vietnam
1	Psychoeducation	Used the words “Common Mental Disorder” to refer to a collective term of depression, anxiety, and stress-related disorders instead of “kufungisisa” (Zimbabwean for depression and anxiety). Removed information about tablets
2	Core competencies	Changed chapter title from “Lay health workers” to “Counselors” Replaced the Zimbabwean CMD^b^ screening SSQ^c^ with DASS-21^d^ for research in Vietnam
3	Mental illness or mental neurological substance use disorders	Removed information about postpartum depression, fatherhood, sexual partners, and family
4	Epilepsy	Removed completely
5	Medication	Kept information about ART^e^ Added information about MMT^f^
6	Belief in supernatural powers	Removed completely
7	HIV and mental health	Added information on HIV transmission through injection Removed information about misunderstandings about church teachings
8	Substance use disorders	Added information on substance use disorders caused by injecting drugs Added information about amphetamine-type stimulants Removed the substance use chemicals that cause bronchodilators Removed alcohol and pregnancy
9	Psychosis	Removed completely
10	Problem-solving therapy	Retained
11	Questionnaires	Replaced Screening CMD by SSQ in Zimbabwe by DASS-21 in study
12	Counseling skills	Retained
13	FB cards	Retained
14	Emotions	Retained
15	Stabilization	Retained
16	Strong emotional reactions	Retained
17	Men’s health-seeking behaviors	Retained
18	Suicide assessment	Replaced suicide assessment of SSQ by the suicide assessment of the study in Vietnam
19	Supervision	Changed monitor titles to the study staffs in Vietnam
20	Home visits	Removed completely
21	Group circle	Removed completely
22	Self-care	Retained
23	Proverbs	Removed completely
N/A	Training overview	Changed training curriculum from 8 days to 5 days. Rewrote new training program according to the virtual format
N/A	Shona training material	Removed completely
N/A	Others	Rewrote the illustrative examples taking information from the qualitative research of step 3 and making it specific to the study population and Vietnamese culture Removed the instructions for using the tablet from the original document because the study in Vietnam used paper-based manual materials

^a^N/A: not applicable.

^b^CMD: common mental disorder.

^c^SSQ: Shona Symptoms Questionnaire.

^d^DASS-21: Depression, Anxiety, and Stress Scale-21 items.

^e^ART: antiretroviral therapy.

^f^MMT: methadone maintenance treatment.

All cases were revised, and the PST case was rewritten. In the original FB, the PST case featured a common problem in the Zimbabwe community, and the main character was a person with an unknown HIV status, a person with SUD, or a woman experiencing domestic violence. The new character in the adaptation was people living with HIV who inject drugs with short-term, solvable problems about employment, family matters, finances, and relationships with neighbors.

### Phase 5: Topical Experts

The first English version was sent to 2 investigators, 2 creators of FB, and 1 expert from HMU for viewing and editing. All of them are associate professors with intensive expertise in the areas of mental health, HIV or AIDS, and substance use. They have led clinical studies and trials on a global scale. Experts advised correcting cases of PST to adjust them to Vietnamese culture. They recommended not changing the in-person counseling procedures and having up to 6 individual counseling sessions for each patient. An FB creator wrote scripts for the counselors to be included in the draft. The creator also advised maintaining the PST structure, with 4 main parts in each counseling session. The 4 main parts were the following: open your mind, make a list of problems, plan to solve the problem according to the Specific, Measurable, Achievable, Realistic, and Timely method, and encourage the clients to implement the plan. The 4 main parts of the PST were implemented into 7 small steps: (1) How does the client deal with problems? (2) How to recognize a problem? (3) How to select a problem, find the goal, and define the problem? (4) How to brainstorm for solutions? (5) How to select a solution? (6) How to make a Specific, Measurable, Achievable, Realistic, and Timely action plan? and (7) Did it work?

### Phase 6: Integration

On October 20, 2021, a meeting was held on the web with 3 content experts, the study team in Vietnam, and 2 investigators. The investigators agreed on continuing to revise the case example of a typical people who inject drugs who encounters common mental problems in their life. These problems should be specific, simple, and solvable within 1 week. For example, “I want to quit using drugs for the next 1 year because next week is my son’s birthday.” The study team agreed to remove the group discussion portion, as it would be ineffective where MMT clinics are far from each other, without finding additional study resources. The study team then synthesized the expert revision to create the second draft in English. Illustrations of Vietnamese people were added to the second English version to make the third version which was sent to 3 content experts and 2 investigators to review and finalize. The third English version was translated into Vietnamese with the same content and images. A Vietnamese topical expert reviewed the translated third version to ensure conceptual equivalency.

## Discussion

### Principal Findings

This study successfully illustrates the process of operationalizing the ADAPT-ITT framework to adapt to a mental health program in Vietnam. The outcome of this study was an adapted FB manual to be used for training and piloting to create the final program manual.

We note that our study departs from the standard ADAPT-ITT framework by selecting an EIP that does not come from the CDC database of EIPs [26] and by adapting a program addressing CMDs rather than HIV prevention per the usual ADAPT-ITT goals [25]. Nevertheless, our study was methodologically aligned with the ADAPT-ITT goals, as we chose a proven, effective program for adaptation. FB improved CMDs among people living with HIV in Zimbabwe and Malawi, and we hope to use FB to address CMDs among people living with HIV on MMT in Vietnam.

The application of the ADAPT-ITT framework for the systematic adaptation of EIP can vary depending on the program, population, and resources [28,30,39]. A key strength of this study is that it used a linear approach to adapt the ADAPT-ITT framework on a small scale [28]. Results of the previous phases provided informative contributions for the next phase [40] in terms of the program procedures and content of the program manual. Other study and adaptation strengths were the translation into Vietnamese and the creation of local illustrations to increase the clarity of the concepts while maintaining the original meaning of the English version.

The results from phase 3 informed the program procedures built upon the existing resources and administrative strength of MMT clinics [40]. As a result of adaptation, the study will choose health care providers and people living with HIV or community members who are trusted by the study participants to work as study counselors. The counselors will be trained and managed closely to ensure the quality of counseling. The counseling locations must be private and safe in MMT clinics. Counseling appointments will be scheduled between counselors and patients in parallel. Standardized criteria of counselors to deliver the program are defined as being understanding and having the trust of the patients but not necessarily having a high level of counseling techniques before training. Instead of using tools and procedures in the original FB program, the study will use questionnaires and protocols relevant to the study participants.

Regarding the content of the manual, the ADAPT-ITT framework guided the appropriate adaptation of the program while maintaining the core components of the PST of the original program throughout the counseling techniques in all program sessions [25,26]. The strength of our adaptation process was that the PST of the original FB fits the goals of reducing CMDs for people living with HIV on MMT in Vietnam; therefore, we did not have to change PST counseling as well as other chapters mentioning CMDs, HIV, and SUD in the original program that address common problems that global and Vietnamese people living with HIV and people who inject drugs encounter [41,42]. Feedback from topical experts informed the study team to alter some contents of the original program to ensure that it was suitable for Vietnamese people living with HIV on MMT and in MMT settings [27,40]. Specifically, unlike the original program conducted in Zimbabwe, we cut content that is not common in Vietnamese culture such as a church, beliefs in supernatural power, and praying together. The study also changed the illustrations from Zimbabwean to Vietnamese. Information from a qualitative study in phase 3 about common health problems faced by people living with HIV on MMT (the results are not reported within the scope of this paper) helped the study team rewrite sample PST cases to reflect typical problems in the key population. In addition, because of the new study with people living with HIV on MMT in Vietnam, we added additional guidelines on HIV, injecting drugs, and SUD to the original program to make it more culturally relevant and relevant to the study population. As recommended by topical experts, we dropped group activities that were proven more adaptive to the Zimbabwean setting and chapters less relevant to the target population (fatherhood, alcohol and pregnancy, epilepsy, and psychosis).

### Limitations

This study has some limitations, the first being that the process of adaptation and production has not yet tested the feasibility and acceptability of the adapted manual. In addition, the semistructured interviews had not presented the original manual to the study participants. Therefore, we did not have comments from them on the content of the manual. Instead, the content of the manual was reviewed and revised in detail according to the study team’s revisions and experts’ comments. There might be challenges such as following the structure of FB counseling sessions, applying relevant counseling skills required in FB, following PST methods for health care, and lay counselors using the adapted manual as a resource material in conducting PST counseling sessions as they first work with this approach. As such, the adapted manual will be used to train counselors and piloted with the study participants of the future RCT to receive feedback to finalize the manual. Study participants and counselors can provide their feedback during the pilot phase. Future RCTs will test the acceptability and fidelity of the finalized adapted program.

### Conclusions

This initial exploratory study demonstrates a successful process of following the ADAPT-ITT to adapt a proven mental health program for people living with HIV on MMT in Vietnam. This study selected and culturally adapted an evidence-informed PST program to improve CMDs among people living with HIV on MMT in Vietnam. The adaptation of the program through qualitative interviews and discussions with stakeholders and study participants and the use of feedback to tailor the program procedures allowed us to identify and preemptively address potential barriers to implementation. The production, integration, and expert input phases were used to tailor the manual to reflect typical manageable problems that people living with HIV on MMT encounter daily. If the adapted FB manual is acceptable and feasible, it may be used in MMT clinics in Vietnam to reduce CMDs for patients on MMT, which would contribute to the effectiveness of drug treatment and ART.

## Abbreviations

ADAPT-ITT: Assessment-Decision-Adaptation-Production-Topical Experts-Integration-Training-Testing
ART: antiretroviral therapy
CDC: Centers for Disease Control and Prevention
CMD: common mental disorder
DASS-21: Depression, Anxiety, and Stress Scale-21 items
EIP: evidence-informed program
FB: Friendship Bench
FGD: focus group discussion
HMU: Hanoi Medical University
IDI: in-depth interview
MMT: methadone maintenance treatment
PST: problem-solving therapy
RCT: randomized controlled trial
SSQ-14: Shona Symptoms Questionnaire
SUD: substance use disorders
